# Unveiling the pharmacological potential of plant triterpenoids in breast cancer management: an updated review

**DOI:** 10.1007/s00210-024-03054-2

**Published:** 2024-04-02

**Authors:** Shaza H. Aly, Abdullah M. M. Elbadry, Ahmed S. Doghish, Heba A. S. El-Nashar

**Affiliations:** 1 Department of Pharmacognosy, Faculty of Pharmacy, Badr University in Cairo, Cairo, 11829 Egypt; 2 Badr University in Cairo Research Center, Badr University in Cairo, Badr City, 11829 Cairo Egypt; 3 Department of Biochemistry, Faculty of Pharmacy, Badr University in Cairo (BUC), Badr City, , 11829 Cairo Egypt; 4 Department of Biochemistry and Molecular Biology, Faculty of Pharmacy, Al-Azhar University, Nasr City, 11231 Cairo Egypt; 5 Department of Pharmacognosy, Faculty of Pharmacy, Ain Shams University, Cairo, 11566 Egypt

**Keywords:** Breast cancer, Cytotoxicity, Mechanism of action, Natural compounds, Triterpenoids

## Abstract

Breast cancer is the most prevalent type of cancer, the fifth leading cause of cancer-related deaths, and the second leading cause of cancer deaths among women globally. Recent research has provided increasing support for the significance of phytochemicals, both dietary and non-dietary, particularly triterpenoids, in the mitigation and management of breast cancer. Recent studies showed that triterpenoids are promising agents in the treatment and inhibition of breast cancer achieved through the implementation of several molecular modes of action on breast cancer cells. This review discusses recent innovations in plant triterpenoids and their underlying mechanisms of action in combating breast cancer within the timeframe spanning from 2017 to 2023. The present work is an overview of different plant triterpenoids with significant inhibition on proliferation, migration, apoptosis resistance, tumor angiogenesis, or metastasis in various breast cancer cells. The anticancer impact of triterpenoids may be attributed to their antiproliferative activity interfering with angiogenesis and differentiation, regulation of apoptosis, DNA polymerase inhibition, change in signal transductions, and impeding metastasis. The present review focuses on several targets, mechanisms, and pathways associated with pentacyclic triterpenoids, which are responsible for their anticancer effects. We could conclude that natural triterpenoids are considered promising agents to conquer breast cancer.

**Graphical Abstract:**

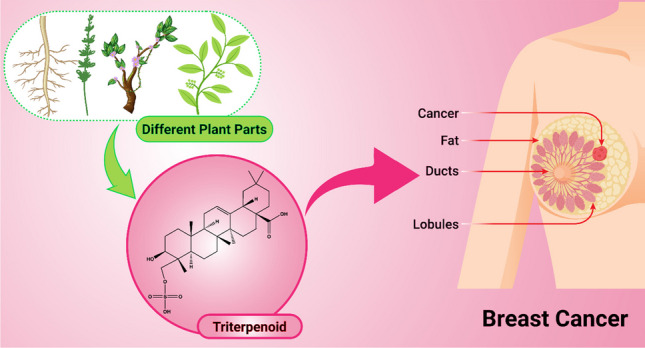

## Introduction

Breast cancer (BC) is one of the deadly diseases that affects women’s lives globally (Askar et al. 2022; Hussein et al. 2024). According to the World Health Organization (WHO), in 2020, The number of women diagnosed with breast cancer worldwide was 2.3 million, while 685,000 died from the disease. As a result, BC is the most prevalent type of cancer, the second leading cause of woman cancer-related deaths globally, and the fifth most common cause of cancer-related deaths overall (Iacoviello et al. 2021; Łukasiewicz et al. 2021). Based on immunohistochemistry (IHC), BC can be categorized into histological and molecular types. Histological types are further classified into invasive and non-invasive types, which are further subdivided into lobular and ductal subtypes, while molecular types are classified based on the presence or absence of molecular markers for human epidermal growth factor 2 (HER2/ ERBB2), progesterone receptor (PR), or estrogen receptor (ER) (Thakur et al. 2023; Weigelt & Reis-Filho 2009).

**Fig. 1:**
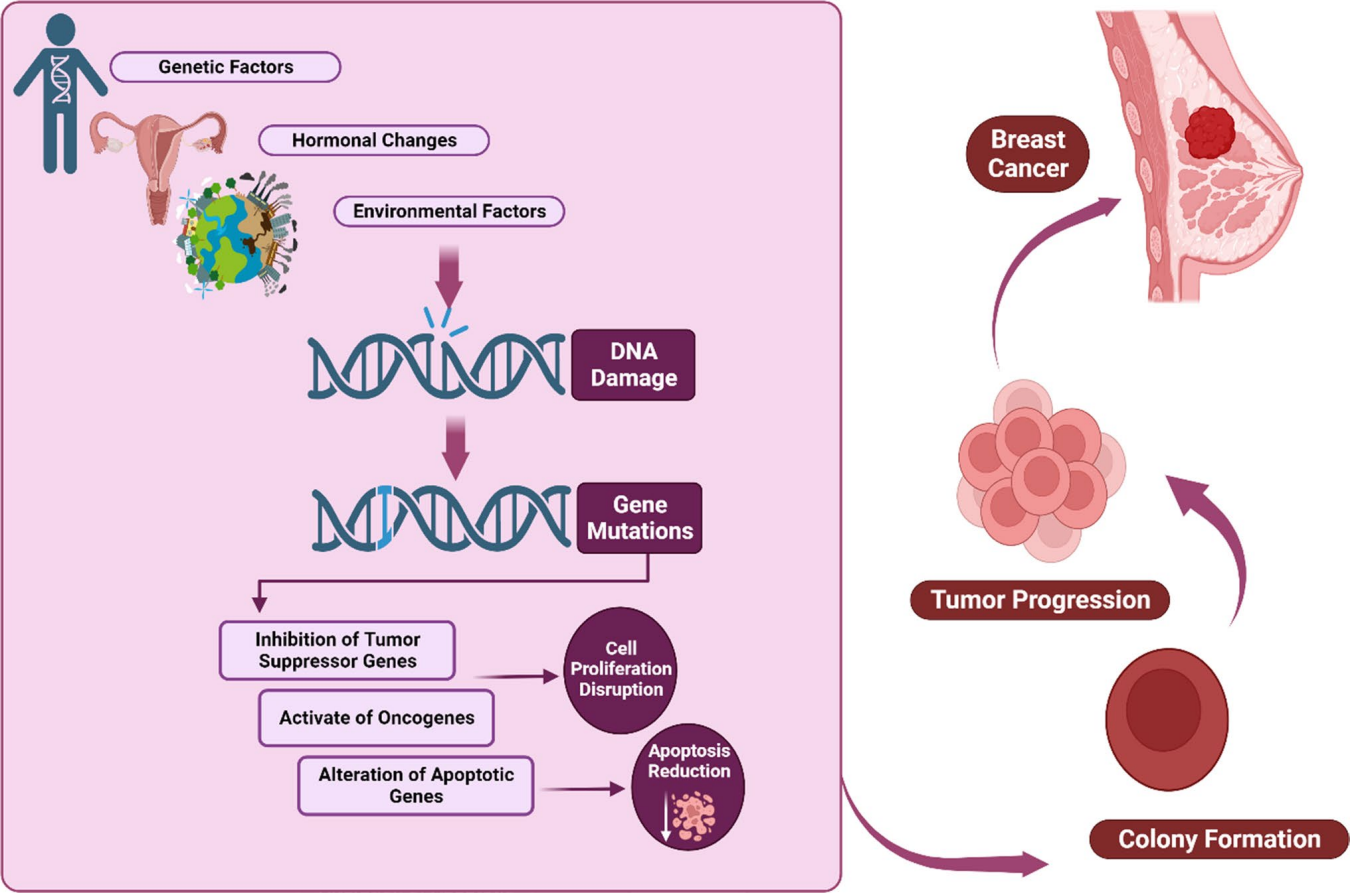
The pathophysiology of breast cancer

Approximately 70% of patients are diagnosed with hormone receptor (HR) positive/HER2 negative breast cancer, while 15 to 20% of patients are diagnosed with HER2 positive breast cancer. The remaining 15% of patients are diagnosed with triple-negative breast cancer (TNBC), characterized by the absence of all three standard molecular markers ER, PR, and HER2. This subtype is associated with a higher likelihood of relapse and is typically treated solely with chemotherapy (Li et al. 2022; Waks and Winer 2019). Tumor elimination and recurrence prevention are the therapeutic goals for nonmetastatic breast cancer. According to the subtype, systemic therapy is established where cases with hormone-receptor-positive tumors acquire endocrine therapy, and chemotherapy is used in a small percentage of cases. Cases with *ERBB2*-positive cancer are treated with *ERBB2*-targeted antibodies or utilization of small-molecule inhibitor treatment in conjunction with chemotherapy. Surgical resection with the inclusion of postoperative radiotherapy is the local therapy for all cases of nonmetastatic breast cancer (Waks and Winer 2019). Figure 1 illustrates the pathophysiology of the breast cancer. DNA damage elevates the frequency of gene mutations and the risk of developing cancer (Basu 2018). This may result from genetic factors, hormonal changes, and environmental factors (Di Sante et al. 2017; Gray et al. 2009; Łukasiewicz et al. 2021). In breast cancer, when enough mutations accumulate in breast cells, the cells eventually transform into tumors, which leads to genomic instability and tumor progression. This may be caused by a variety of types of mutations, such as mutations in tumor suppressor genes and apoptotic genes like BRCA1, BRCA2, and P53, which become oncogenes after mutation (Alharbi et al. 2022; Lim et al. 2009; Lin et al. 2022; Moon et al. 2023).

Due to the ineffectiveness of conventional therapy, the rate of tumor relapse of breast cancer is quickly increasing where it is manifested by variable morphological appearances, molecular characteristics, and behavior. Notwithstanding the advancements in cancer research, detection, diagnosis, and treatment, there is still a long way to go especially since many patients’ treatment has been hampered by the adverse consequences of radiotherapy and chemotherapy, along with the phenomenon of drug resistance (El-Nashar, Aly, et al. 2021a, b; Ghate et al. 2014).

Plants provide a wealthy source of natural compounds with diverse pharmacological properties (Abd El-Ghffar et al. 2017; Abdelazim et al. 2024; Abdelghffar et al. 2021; Goher et al. 2023; Mia et al. 2023). During the last years, the discovery of several bioactive compounds has been explored in plants as chemopreventive and therapeutic agents as well as to reduce medication toxicity and resistance (Aly et al 2020a; Aly et al. 2021; Aly et al. 2023a; Elebeedy et al. 2023; Otsuki and Li 2023). Besides, the need for natural treatments to limit tumor progression, improve quality of life, and extend survival is urgently needed (Aly, Elissawy, Fayez, et al. 2020a, b, c; Dennis et al. 2009; El-Nashar et al. 2021a, b; Song et al. 2020). Terpenoids including terpenes and isoprenoids are the most abundant secondary metabolites in plants (Aly et al. 2023b; Aly, Elissawy, Mahmoud, et al. 2023b, c, d, e, a; Aly, Elissawy, Salah, et al. 2023b, c, d, e, a; Aly et al. 2022; Yazaki et al. 2017). They are classified into different categories based on the number of isoprene units, such as monoterpenes, sesquiterpenes, diterpenes, sesterterpenes, triterpenes, tetraterpenes, and polyterpenes (Abdelghffar, El-Nashar, et al. 2022a, b; Abdelghffar, Mostafa, et al. 2022a, b; Aly et al. 2023b, c, d, e, a). Triterpenoids, a subclass of terpenoids, have lately emerged as a unique category of phytochemicals with multifunctional anticancer properties, as evidenced by preclinical results (Ads et al. 2022; Bishayee et al. 2011; Kim et al. 2016; Liang et al. 2014; Rabi and Bishayee 2009; Yuan et al. 2012). For example, Paclitaxel (Taxol®), a diterpene derived from *Taxus brevifolia*, had been approved as a commercial anti-neoplastic treatment in 1993 signaling the terpenoids’ imminent entry into the anti-cancer field, with the variability of triterpenoids structures making them a potential source for breast cancer treatment (Anand and Francis 2016).

**Fig. 2:**
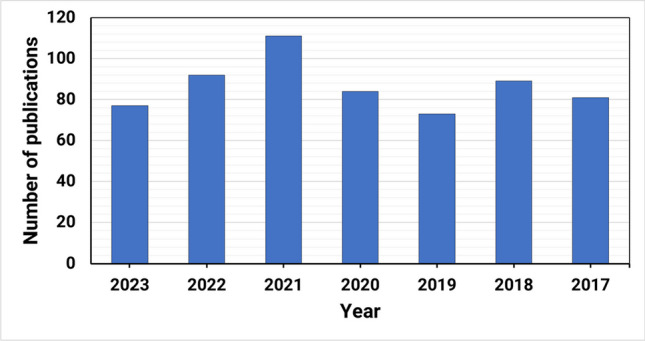
Number of publications on “Plant triterpenoids and breast cancer” in PubMed, arranged by decade from 2017 to 2023

**Fig. 3:**
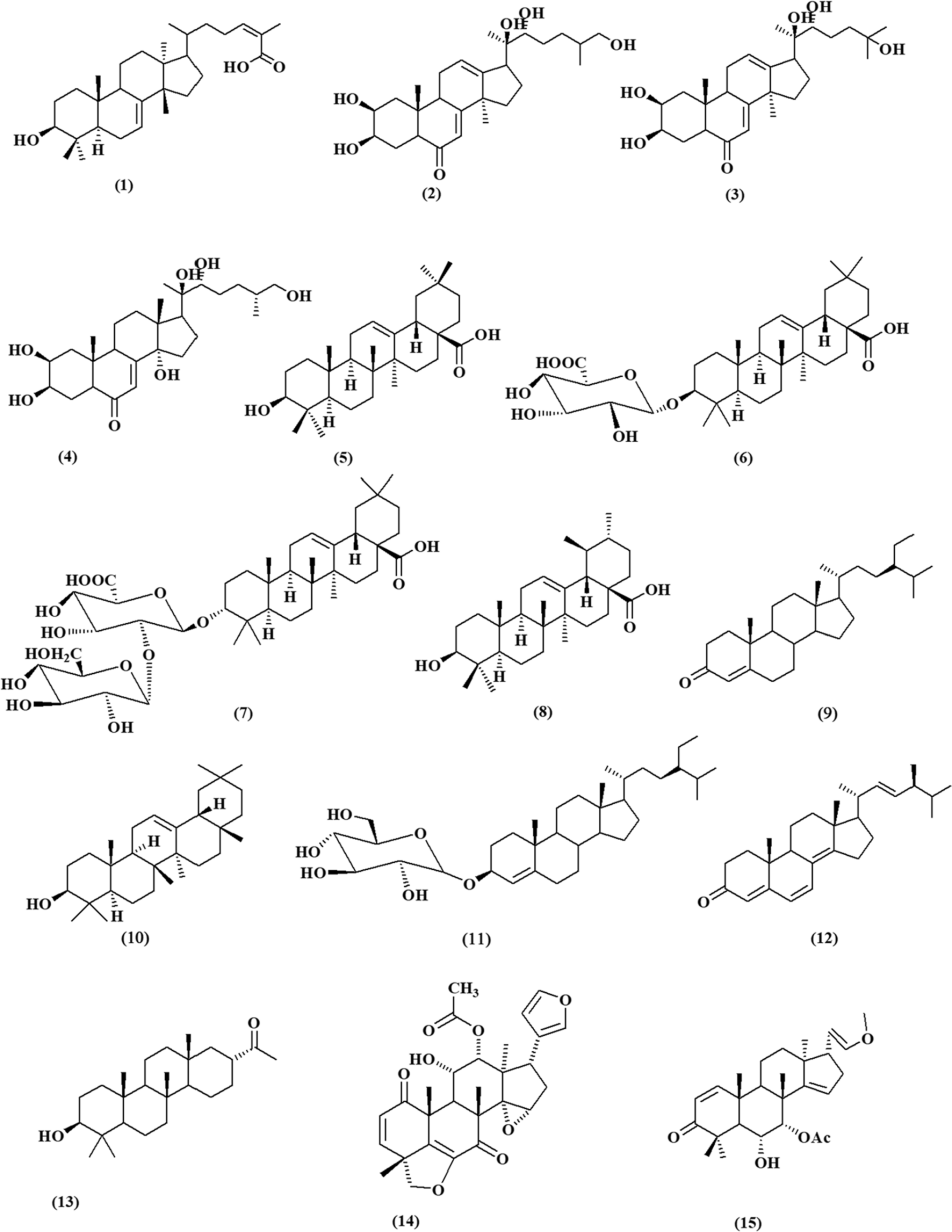
Chemical structures of isolated triterpenoids with potential cytotoxicity towards breast cancer cells **(1-15)**

**Fig. 4:**
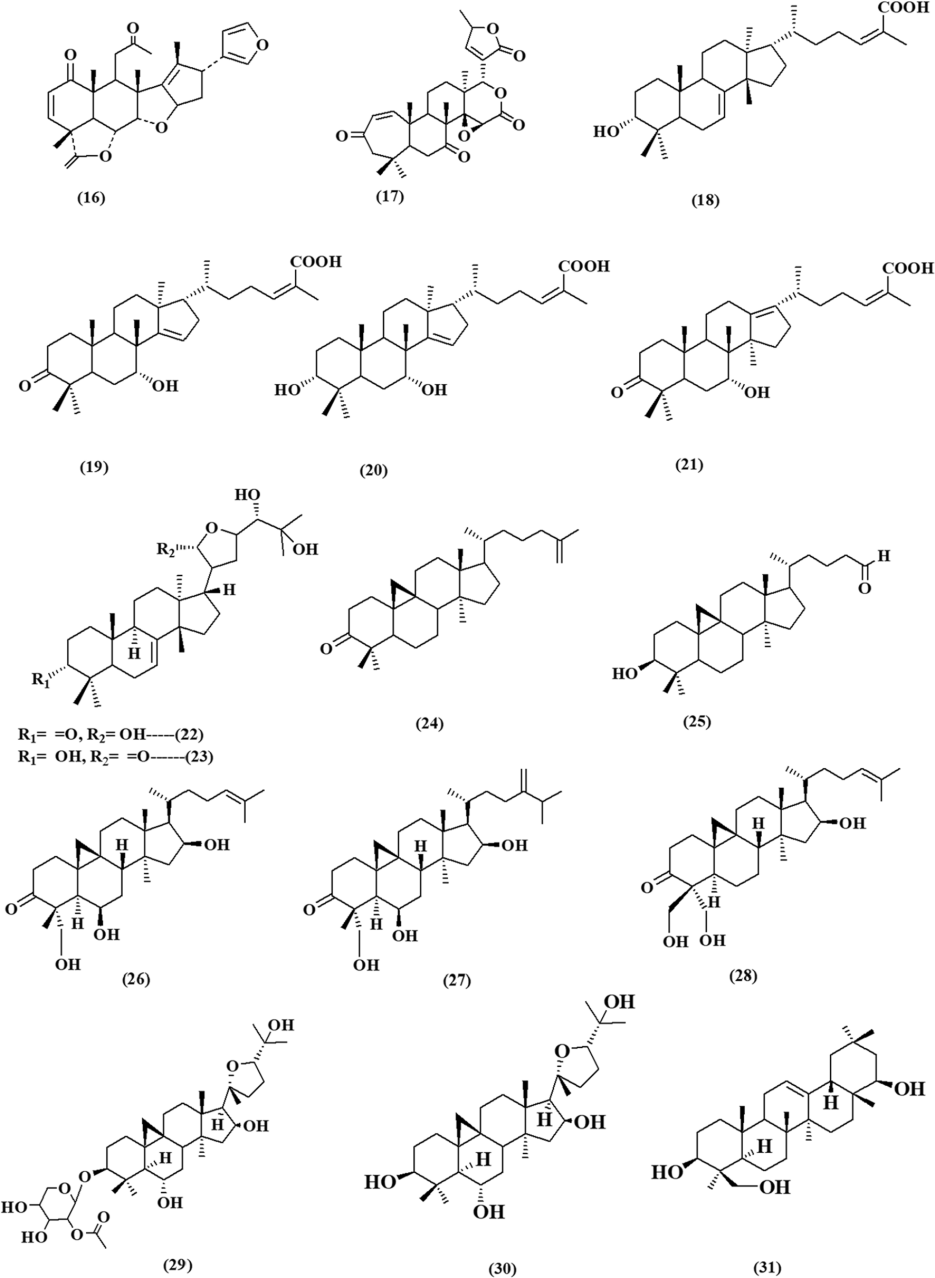
Chemical structures of isolated triterpenoids with potential cytotoxicity towards breast cancer cells **(16-31)**

**Fig. 5:**
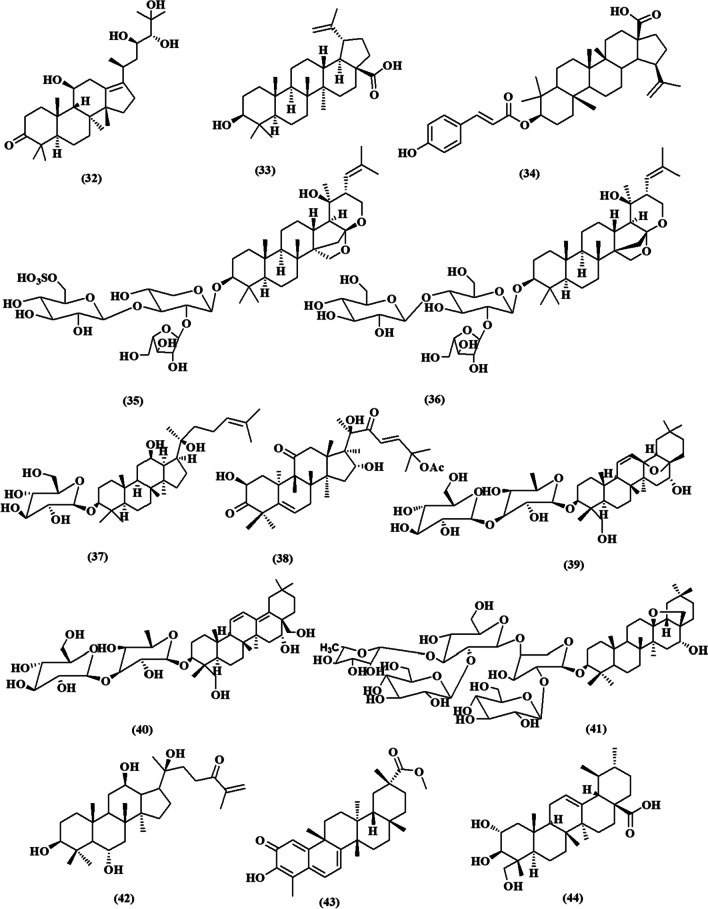
Chemical structures of isolated triterpenoids with potential cytotoxicity towards breast cancer cells **(32-44)**

**Fig. 6:**
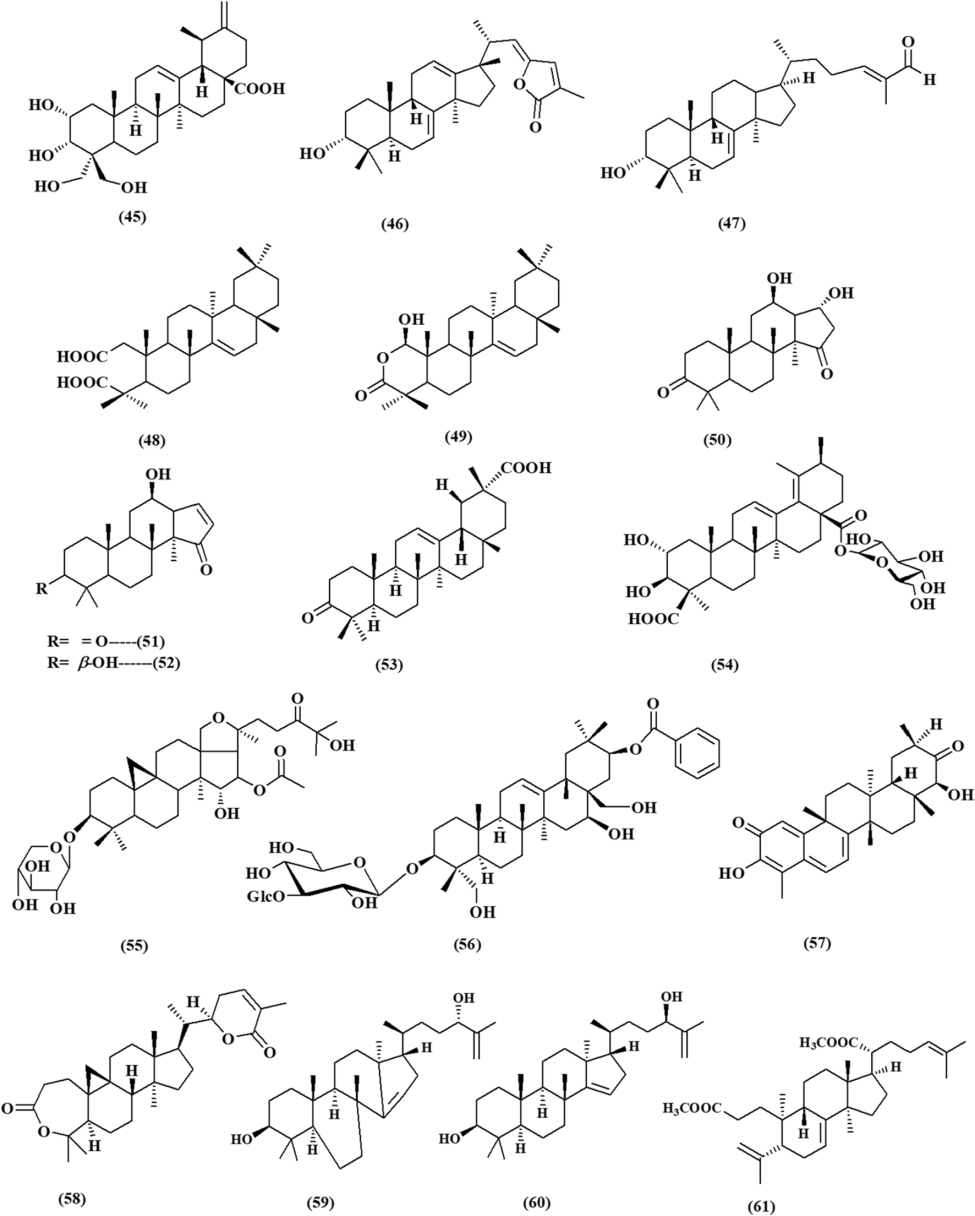
Chemical structures of isolated triterpenoids with potential cytotoxicity towards breast cancer cells **(45-61)**

**Fig. 7:**
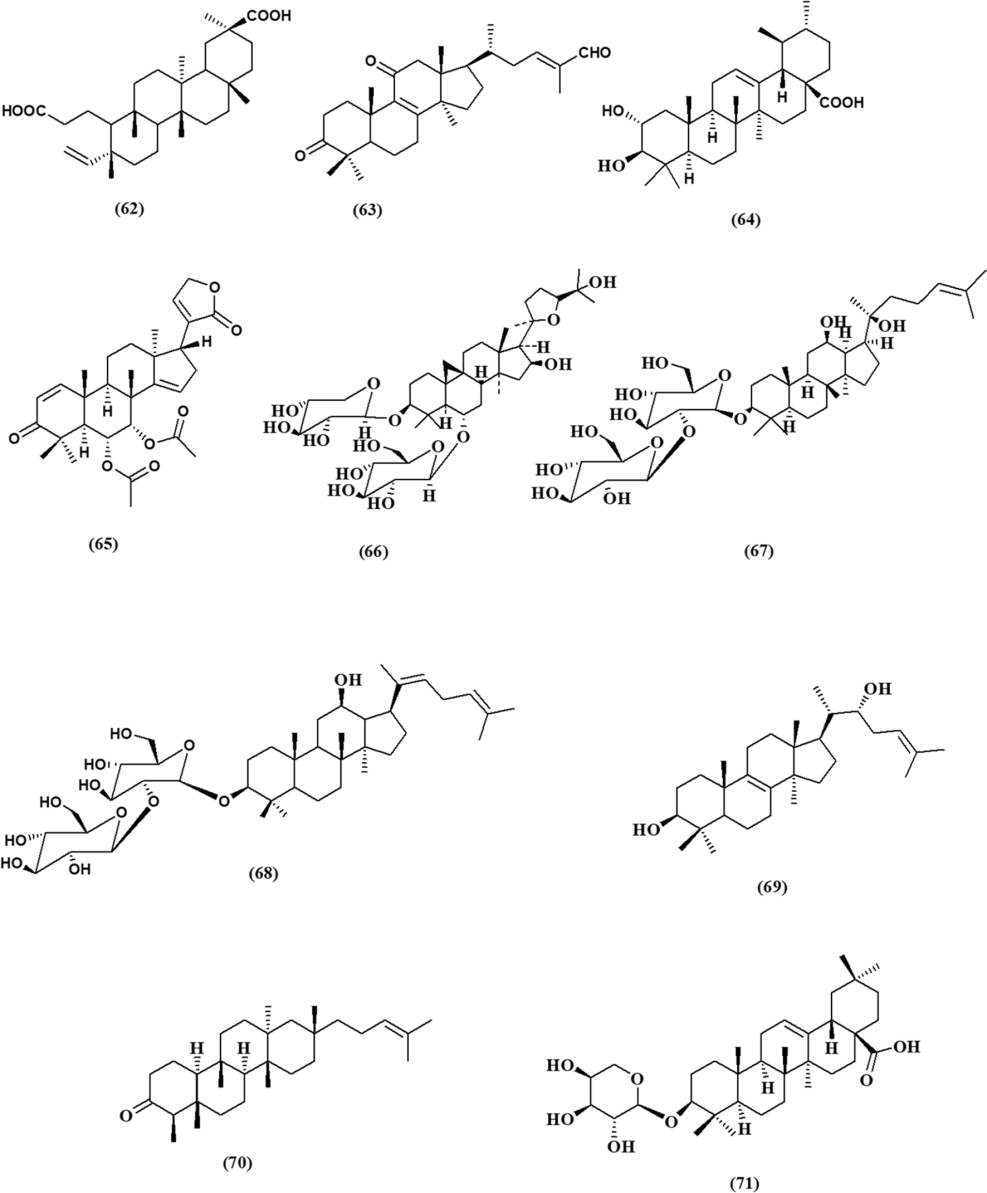
Chemical structures of isolated triterpenoids with potential cytotoxicity towards breast cancer cells **(62-71)**

This review contemplated the latest findings on the cytotoxic activities of plant-derived triterpenoids during the period of (2017–2023) (Fig. 2). The relevant data has been collected from databases including PubMed, Google Scholar, ScienceDirect, and Web of Knowledge. The details of the chemical structures of the studied compounds (Figs. 3, 4, 5, 6, and 7) were obtained from PubChem and drawn using PerkinElmer ChemDraw Professional version 16.0 software. We aim to conduct an updated convenient review that will be of scientific merit to both researchers and readers who are interested in the cytotoxic implications of natural triterpenoids.

## In vitro studies of plant triterpenoids on the management of breast cancer

The effectiveness of plant triterpenoids isolated from different plant species has been evaluated using different techniques as MTT, WST- 1, and SRB assays against various breast cancer cell lines including MCF-7, MDA-MB-231, T-47D, BT-549, BT-474, and SK-BR-3 (Choodej & Pudhom 2020; Liu et al. 2019; Muthoni et al. 2021; Trinh et al. 2019). The triterpenoids that have been shown to have potential *in vitro* cytotoxicity towards breast cancer cell lines are outlined in (Table 1).

**Table 1:** Plant triterpenoids with potential *in vitro* cytotoxicity towards breast cancer

Compound name	Plant source	IC_50_	Breast cancer cell line	Refs
Masticadienolic acid (1)	*Dysoxylum excelsum* bark (Meliaceae)	3.5 µM	MCF-7	Zainuddin et al. (2020)
Achyranthesterone B (2)	*Achyranthes bidentata* Blume roots (Amaranthaceae)	11.52 ±1.33 µM	MCF-7	Wang et al. (2014)
Stachysterone A (3)		29.56 ± 2.54 µM		
25-R Inokosterone (4)		20.89 ± 3.20 µM		
Oleanolic acid (5)		11.00 ± 3.87 µM		
Deglucose chikusetsu saponin IVa (6)		10.54 ± 1.02 µM		
Zingibroside r1 (7)		21.00 ± 2.67 µM		
Oleanolic acid (5)	*Dracocephalum heterophyllum* above-ground parts (Lamiaceae)	2.63 ± 0.78 µM	T-47D	Numonov et al. (2020)
		3.08 ± 0.83 µM	SK-Br-3	
Ursolic acid (8)		1.63 ± 0.62 µM	T-47D	
		6.62 ± 1.09 µM	MCF-7	
*β*-Sitosterol (9)	*Vicia monantha subsp. monantha Retz.* (Fabaceae)	15.42 µg/mL	MCF-7	El-Halawany et al. (2019)
*β*-Amyrin (10)		10.08 µg/mL		
*β*-sitosterol-3-O-*β*-D-glucopyranoside (11)		11.34 µg/mL		
22-tetraen-3-one (12), (22*E*, 24*S*)-Ergosta-4,6,8 (14)	*Selaginella moellendorffii,* Hieron (Selaginellaceae)	19.30 ± 0.8 µM	MDA-MB-231	Wu et al. (2020)
		24.2 ± 4.9 µM	MCF-7	
Perisomalien A (13)	*Periploca somaliensis* fruits (Asclepiadaceae)	19.2 mM	MCF-7	Jabal et al. (2020)
*β*-sitosterol-3-O-*β*-D-glucopyranoside (11)				
Walsucochinone C (14)	*Walsura cochinchinensis* bark (Meliaceae)	16.4 ± 0.2 µM	MCF-7	Trinh et al. (2019)
Nimonol (15)	*Chisocheton pentandrus* (Blanco) stem bark (Meliaceae)	22.03 ± 0.026 µM	MCF-7	Supratman et al. (2020)
Nimbolide (16)	*Azadirachta indica* (Meliaceae)	1.97 µM	MDA-MB-231	Pooladanda et al. (2018)
		5.04 µM	MCF-7	
Kihadanin B (17)	*Araliopsis soyauxii* Engl. roots (Rutaceae)	7.79 ± 0.57 µM	MDA-MB-231-*pcDNA*	Fotso et al. (2020)
		8.04 ± 0.82 µM	MDA-MB-231-*BCRP*	
Chisopaten A (18)	*Chisocheton patens* Blume Bark (Meliaceae)	4.01 ± 0.008 µM	MCF-7	Supratman et al. (2019)
Chisopaten B (19)		6.98 ± 0.008 µM		
Chisopaten C (20)		4.33 ± 0.009 µM		
Chisopaten D (21)		9.23 ± 0.008 µM		
Melianodiol (22) and Indicalilacol B 19 (23)	*Chisocheton pentandrus* (Blanco) Merr. stem bark (Meliaceae)	16.84–20.98 µM µM	MCF-7	Salam et al. (2021)
Cycloschimperols B (24)	*Euphorbia schimperi* aerial parts (Euphorbiaceae)	4.70 ± 0.1 µM	MCF-7	Banjar et al. (2019)
Cycloart-25-en-3-one (25)		2.10 ± 0.01 µM		
Neriifolins A (26)	*Euphorbia neriifolia* Linn leaves (Euphorbiaceae)	13.14 ± 1.12 µM	MCF-7	Choodej and Pudhom (2020)
		27.71 ± 1.44 µM	MDA-MB-231	
Neriifolins B (27)		7.12 ± 0.85 µM	MCF-7	
		21.12 ± 1.33µM	MDA-MB-231	
Neriifolins C (28)		9.50 ± 0.98 µM	MCF-7	
		26.77 ± 1.43 µM	MDA-MB-231	
20(*R*),24(*S*)-Epoxycycloartane-3*ꞵ*,6*α*,16*ꞵ*,25-tetraol-3*ꞵ*-*O*-D-(2-*O-*acetyl) xylopyranoside (29)	*Caragana sukiensis* aerial parts (Fabaceae)	2.931 µm	MCF-7	Reddy et al. (2017)
Cyclosiversigenin (30)		1.778 µm		
Soyasapogenol B (31)		2.328 µm		
Alisol A (32)	*Alisma orientale* rhizomes (Alismataceae)	Dose of 5 µM	MDA-MB-231	Lou et al. (2019a, b), Shi et al. (2020).
Betulinic acid (33)	*Betula papyrifera* bark (Betulaceae)	19.06 µM	MCF-7	Jiao et al. (2019)
3-O-(E)-*p*-coumaroylbetulinic acid (34)	*Strychnos vanprukii* Craib (Loganiaceae), and *Cornus florida* L. (Cornaceae)	5.884 µM	MDA-MB-231	Kushwaha et al. (2020)
		2.708 µM	T-47D	
Bacopasides II (36)	*Bacopa monnieri* (Plantaginaceae)	18 µM	MDA-MB-231	Palethorpe et al. (2019).
		19 µM	MCF7	
		16 µM	BT-474	
Ginsenoside Rh2 (37)	*Panax ginseng* (Araliaceae)	Suppressed cell proliferation up to 46%	MCF-7	Jeong et al. (2019)
Cucurbitacin B (38)	*Momordica charantia* L. and *Trichosanthes cucumerina* (Cucurbitaceae)	15.89 µM	MDA‐MB‐231	Liang et al. (2019)
		6.177 µM	SKBR‐3	
Saikosaponin D (39)	*Bupleurum chinense* DC. Roots (Apiaceae)	9.9 ± 0.24 µM	MDA‑MB-231	Ma et al. (2019)
Saikosaponin b2 (40)		Dose of 5 µM	MCF-7	
AG8 (41)	*Ardisia gigantifolia* stapf. rhizomes (Primulaceae)	3.80 µM	MDA-MB-231	Mu et al. (2020a, b)
		0.73 µM	BT-549	
		1.38 µM	MDA-MB-157	
3*β*, 6*α*, 12*β*, 20 (*S*)-tetrahydroxydammar-24-one-25-ene (42)	*Panax notoginseng* (Burk.) roots (Araliaceae)	16.78 ± 0.32 µM	MCF-7	Shang et al. (2019)
Pristimerin (43)	Celastraceae and Hippocrateaceae families	IC_50_ values ranging between 0.38 and 1.75 µM	MCF-7 and MDA-MB-231 and MCF-7s	
Asiatic acid (44)	*Actinidia valvata* roots (Actinidiaceae)	Dose of 50 µM	MDA-MB-231	Gou et al. (2020)
2*α*, 3*α*, 23, 24-tetrahydroxyursa-12, 20(30)-dien-28-oic acid (45)	*Actinidia chinensis* Planch (Actinidiaceae)	45.94 ± 3.62 µM	MCF-7	Zhang et al. (2018a, b)
Neoabieslactone L (46)	*Abies faxoniana* (Pinaceae).	7.5 µM	MCF-7	Viet Ho et al. (2018)
Abiesatrine Q (47)		22.20 µM		
2, 3-*seco*-taraxer-14-en-2, 3-bioic acid (48)	*Euphorbia alatavica (*Euphorbiaceae)	22.3±3.1 µM	MCF-7	Rozimamat et al. (2017).
3-Hydroxy-4, 4, 8,14-tetramethyl-18-norpregnan-20-one (49)		8.6 ± 3.5 µM		
Vibusambucin A (50)	*Viburnum sambucinum* leaves (Adoxaceae)	5.7 µM	MCF-7	Nguyen et al. (2017).
12*β*-hydoxy-3,15-dioxo-20, 21, 22-23, 24,25, 26, 27-octanordammanran (51)		5.30 µM		
Hupehenol A (52)		5.10 µM		
Katononic acid (53)	*Nuxia oppositifolia* aerial parts (Stilbaceae)	18.25 µM	MDA-MB-231	Al-Massarani et al. (2017)
Chestnoside B (54)	*Castanea sativa* (Fagaceae)	12.3 µM	MCF-7	Pérez et al. (2017).
Soulieoside O (55)	*Souliea vaginata* rhizomes (Ranunculaceae).	9.3±2.10 µM	MDA-MB231	Wu et al. (2017).
Hirsutosid B (56)	*Glochidion hirsutum* leaves (Phyllanthaceae)	4.7 ± 0.6 mM	MCF-7	Thang et al. (2017)
Tingenin b (57)	*Mytenus* sp. root bark (Celastraceae)	2.38 µM	MCF-7	Cevatemre et al. (2016)
Schisanlactone B (58)	*Kadsura coccinea* stems (Schisandraceae)	2.38 µM	MDA-MB231	Tram et al. (2021)
Lugardstatin 1 (59)	*Monadenium lugardae* roots and aerial parts (Euphorbiaceaewas).	0.79 µM	MCF-7	Pettit et al. (2016)
Lugardstatin 2 (60)		0.68 µM		
Leplaeric acid B (61)	*Leplaea mayombensis* roots (Meliaceae)	55.0 ± 7µM	MDA-MB231	Sidjui et al. (2017)
secofriedelanophyllemblicine (62)	*Phyllanthus emblica* L. roots (Phyllanthaceae),	Reduction in cell viability by 70.65% after treatment with 2 µM.	MCF-7	Wang et al. (2022a, b)
Endertiin A (63)	Fruit bodies of the mushoom *Humphreya endertii*	71.16 ± 6.25 µg/mL	MCF-7	Quang et al. (2022)
Corosolic acid (64)	*Lagerstroemia speciosa* Leaves (Lythraceae)	20.12 µM and 28.50 µM	MDA-MB-231 and MCF7	Jasim et al. (2023)
Lasiocarpine A (65)	*Chisocheton lasiocarpus* Fruits (Meliaceae)	IC_50_ = 43.38 µM	MCF-7	Katja et al. (2023)

Figure 3 shows the chemical structures of triterpenoids that have been identified and could potentially be cytotoxic to breast cancer cells **(1–15)**. Masticadienolic acid **(1)**, is a triterpenoid that has been isolated for the first time from the stem bark of *Dysoxylum excelsum* (Meliaceae). It demonstrated *in vitro* cytotoxic activity using the MTT assay with an IC_50_ value of 3.5 µM against MCF-7 breast cancer cell lines (Zainuddin et al. 2020). It exhibited strong cytotoxic effects based on molecular docking studies as it is a tetracyclic triterpenoid typical compound with carboxylic acid and olefinic moieties which could strengthen the inhibition activity against the aromatase targeting CYP19A1 of MCF7 cell lines (Prakash et al. 2014).

The roots of *Achyranthes bidentata* Blume (Amaranthaceae) were subjected to separation using network pharmacology analysis. The root extract revealed the isolation of six triterpenoids and investigated for inhibitory effects on MCF-7 cells using MTT assay. It was found that achyranthesterone B **(2)** exhibited IC_50_= 11.52 ±1.33 µM, stachysterone A **(3)** with IC_50_ = 29.56 ± 2.54 µM, 25-R inokosterone **(4)** with IC_50_ = 20.89 ± 3.20 µM, oleanolic acid **(5)** with IC_50_ = 11.00 ± 3.87 µM, deglucose chikusetsu saponin Iva **(6)** with IC_50_ = 10.54 ± 1.02 µM and zingibroside r1 **(7)** with IC_50_ =21.00 ± 2.67 µM. All isolated compounds **(2–7)** exhibited notable cytotoxicity against breast cancer cells and had potent inhibitory effects on the production and release of nitric oxide (NO) and tumor necrosis factor-α (TNF-α) in macrophage cells induced by LPS. Compound (**6**) showed the most inhibitory action on NO and TNF-α with IC_50_ values of 11.20 ± 1.22 µM and 16.80 ± 1.54 µM, respectively (Ju et al. 2020). The cytotoxic activity of the compound **(6)** can be attributed to its structure, specifically the glycosidation at C-3 and the free carboxyl substitution at C-28. These structural features are crucial for its anti-tumor and anti-inflammatory effects, as demonstrated by the structure-activity relationships (SARs) of oleanane-type triterpenoids. Conversely, the elongation of the sugar chain at position C-3, as shown in compound **(7)**, would result in a reduction of the cytotoxic action (Wang et al. 2014).

From the above-ground parts of *Dracocephalum heterophyllum* Benth. (Lamiaceae), ursolic acid **(8)** (71.9%) and oleanolic acid **(5)** (18.1%) were identified as the major components of its triterpenoidal extract using GC/MS analysis. The combined purified triterpenoid extract from *D. heterophyllum* and its two main components exhibited a notable cytotoxic effect on three different human breast cancer cell lines (SK-Br-3, T-47D, and MCF-7) by using MTT assay. They showed significant reductions in cell proliferation where the total triterpenoids extract showed the highest inhibition activity against the SK-Br-3 cancer cells as compared to pure ursolic acid **(8)**, and oleanolic acid **(5)** with IC_50_ value of 5.91 ± 0.98 µM. The median inhibitory concentration values declared that proliferation and growth of T-47D cells were highly affected by compounds **(8)** and **(5)** with an IC_50_ value of 1.63 ± 0.62 and 2.63 ± 0.78 µM, respectively. Besides, compound **(8)** demonstrated notable inhibition of cell growth in MCF-7 cells, with IC_50_ values of 6.62 ± 1.09 µM. Additionally, oleanolic acid **(5)** had cytotoxic effects against SK-Br-3 cancer cells, with an IC_50_ value of 3.08 ± 0.83 µM. (Numonov et al. 2020). Another report by Mandal et al. Compound **(8)** shows the ability to suppress breast cancer stem-like cells (CSCs) by stimulating the activity of caspase 3/7 and increasing the intracellular level of reactive oxygen species (ROS) in UA-treated CSCs, resulting in a reduction in mitochondrial membrane potential (Mandal et al. 2021). Also, a recent study has demonstrated that ursolic acid (8) has the ability to enhance the sensitivity of MCF-7 and MDA-MB-231 cells to epirubicin, a well-known treatment for breast cancer. This enhancement is achieved by modulating the autophagy pathway through the up-regulation of the expression of autophagy-related proteins Beclin-1, LC3-II/LC3-I, Atg5, and Atg7. Additionally, it reduces the expression levels of PI3K and AKT. These findings suggest that the potential mechanism of action involves the regulation of the class III PI3K(VPS34)/Beclin-1 pathway and the PI3K/AKT/mTOR pathway (Wang et al. 2022a, b).

Ragasa et al. reported that the leaves and twigs of *Wrightia pubescens* (Apocynaceae) are rich in triterpenoids such as ursolic acid **(8)**, oleanolic acid **(5),** and *β*-sitosterol **(9)**, where oleanolic acid **(5)** is a promising cytotoxic drug with an IC_50_ value of 10.99 µM compared to zeocin (IC_50_=4.17 µM; a known anticancer drug) (De Los Reyes et al. 2018; Ragasa et al. 2014).

A recent study by Xu et al. revealed the effectiveness of oleanolic acid **(5)** against MDA-MB-231 cells with an IC_50_ value of 28.02 µg/mL as compared to olaparib an IC_50_ value of 51.73 µg/mL (Xu et al. 2022).

From *Vicia monantha *subsp*. monantha* Retz*.* (Fabaceae) cytotoxic compounds were isolated and evaluated by MTT assay. They showed promising cytotoxicity against breast cancer MCF-7 cells where, *β*-sitosterol **(9)**, *β*-amyrin **(10)**, *β*-sitosterol-glucoside **(11)** showed IC_50_ values of 15.42, 10.08, and 11.34 µg/mL, respectively (El-Halawany et al. 2019).

Two isolated tetracyclic triterpenoids isolated from the whole herb of *Selaginella moellendorffii* Hieron (Selaginellaceae) namely, (22*E*, 24*S*)-Ergosta-4,6,8(14), 22-tetraen-3-one **(12)** and* β*-sitosterol **(9)** showed moderate cytotoxic activities *in vitro* against human breast adenocarcinoma cell lines MDA-MB-231 and MCF-7 using MTT assay. (22*E*, 24*S*)-Ergosta-4,6,8(14), 22-tetraen-3-one **(12)** showed IC_50_ values of 19.30 ± 0.8 and 24.2 ± 4.9 µM against MDA-MB-231 and MCF-7, respectively. While, *β*-sitosterol showed IC_50_ values of 32.0 ± 1.4 and 24.6 ± 5.3 µM against MDA-MB-231 and MCF-7 cells (Wu et al. 2020).

A study conducted on the methanol extract of *Periploca somaliensis* fruits (Asclepiadaceae) discovered a novel scalarane sesterterpene named perisomalien A **(13)**, in addition to previously identified triterpenoids: lupeol acetate, *β*-amyrin, cycloart-23Z-ene-3β,25-diol, and *β*-sitosterol-3-O-*β*-D-glucopyranoside. Cycloart-23Z-ene-3*β*,25-diol demonstrated the highest cytotoxicity among the tested compounds, as shown by the sulforhodamine B (SRB) assay. Its IC_50_ value against MCF-7 cells was 9.0 mM, whereas doxorubicin had an IC_50_ value of 0.18 mM whereas perisomalien A **(13)** and *β*-sitosterol-3-O-*β*-D-glucopyranoside **(11)** had moderate effects with the same IC_50_ value of 19.2 mM (Jabal et al. 2020).

Limonoids are highly oxygenated modified triterpenes, from the bark of *Walsura cochinchinensis* (Meliaceae) a new limonoid walsucochinone C **(14)** was subjected to isolation and subsequently assessed for its cytotoxic effects on MCF-7 human breast cancer cells *in vitro* utilizing the SRB assay. Walsucochinone C **(14)** demonstrated the strongest effect among the isolated compounds with an IC_50_ value of 16.4 ± 0.2 µM. The correlation between the structure and activity of limonoid triterpenoids suggests that the existence of an acetoxy group at C-12 enhances the activity compared to a 2-methylbutyryloxy group. Additionally, the presence of unsaturation at C-5/C-6 further increases the activity (Trinh et al. 2019).

Another known limonoid, nimonol **(15)** isolated from the stem bark of *Chisocheton pentandrus* (Blanco) (Meliaceae) demonstrated strong toxicity against the MCF-7 breast cancer cell line with an IC_50_ = 22.03 ± 0.026 µM. The activity of the compound also showed a correlation with the SARs of limonoids. The inclusion of a furan ring and an acetyl group is crucial for the cytotoxic activity of the limonoid structure, as previously demonstrated (Supratman et al. 2020).

Figure 4 shows the chemical structures of triterpenoids that have been identified and could potentially be cytotoxic to breast cancer cells (**16–31**).

Nimbolide **(16)** is a limonoid tetranortriterpenoid obtained from *Azadirachta indica* (Meliaceae) (Bodduluru et al. 2014). The compound **(16)** demonstrated potent inhibition of MDA-MB-231 and MCF-7 cell growth, with IC_50_ values of 1.97 and 5.04 µM, respectively. It effectively inhibited the advancement of the cell cycle and cell survival by reducing the mitochondrial membrane potential. This was achieved by simultaneously reducing the expression of Bcl-2 and modifying the expression of Bax, caspases, HDAC-2, and H3K27Ac. In addition, it triggered programmed cell death in breast cancer cells by epigenetic alterations by upregulating Beclin 1 and LC3B while downregulating p62 and mTOR protein expression (Pooladanda et al. 2018).

Phytochemical investigation of roots of *Araliopsis soyauxii* Engl. (Rutaceae) led to the isolation of limonoid kihadanin B **(17)** and its cytotoxicity was tested using a resazurin reduction assay (RRA). It showed potent cytotoxicity towards MDA-MB-231-*pcDNA* breast adenocarcinoma cells and MDA-MB-231-*BCRP* breast cancer resistance protein with IC_50_ values 7.79 ± 0.57 µM and 8.04 ± 0.82 µM, respectively. Besides, The positive control, doxorubicin, exhibited IC_50_ values of 0.13 ± 0.01 µg/mL and 0.79 ± 0.08 µg/mL, respectively. Kihadanin B mechanism related to its apoptosis induction by caspase activation, MMP alteration, and enhanced ROS production (Fotso et al. 2020).

A new set of triterpenoids, Chisopaten (A-D) **(18–21)**, were isolated and assessed for their cytotoxic effects on MCF-7 breast cancer cells using the MTT assay. These triterpenoids were obtained from the bark of *Chisocheton patens* Blume, a plant belonging to the family Meliaceae. Two of them Chisopaten A **(18)** and C **(20)** exhibited the most potent cytotoxicity with IC_50_ values of 4.01 ± 0.008 and 4.33 ± 0.009 µM, respectively. Their strong activity could be attributed to the SARs where, the hydroxyl, olefinic, carbonyl, and methyl group positions are playing a vital role in the cytotoxic activity of the isolated compounds. Besides, the carbonyl group in Chisopaten B **(19)** and D **(21)** decreased the activity than in Chisopaten A and C. Furthermore, the presence of a methyl group at C-13 and an olefinic group at C-14/C-15 demonstrated that Chisopaten B had a more potent cytotoxic effect on the MCF-7 breast cancer cell line compared to Chisopaten D. The IC_50_ values for Chisopaten B and Chisopaten D were measured to be 6.98 ± 0.008 µM and 9.23 ± 0.008 µM, respectively (Supratman et al. 2019).

In another study conducted by Salam et al., pentandrucines (A-K) and triterpenoids were isolated from the stem bark *n*-hexane extract of *Chisocheton pentandrus* (Blanco) Merr. (Meliaceae). The compounds pentandrucine J, pentandrucine K, melianodiol **(22)**, and indicalilacol B 19 **(23)** exhibited the highest activity against MCF-7 breast cancer cells, with IC_50_ values ranging from 16.84 to 20.98 µM. Among them, melianodiol **(22)** demonstrated the most potent cytotoxicity, with an IC_50_ value of 16.84 µM, which was comparable to that of Cisplatin (13.2 µM). It has been demonstrated that the inclusion of a lactone ring and a diol group in the side chain enhances cytotoxic activity (Salam et al. 2021).

A new compound called cycloschimperols B **(24)**, which is a cytotoxic cycloartane triterpenoid, has been isolated from the aerial parts of *Euphorbia schimperi* (Euphorbiaceae). It was found with a previously known compound called cycloart-25-en-3-one **(25)**. Both compounds exhibited strong cytotoxicity against MCF-7 cell lines, as shown by the SRB assay. The IC_50_ values for the compounds were 4.70 ± 0.1 µM and 2.10 ± 0.01 µM, respectively (Banjar et al. 2019).

Choodej and Pudhom reported three novel cycloartane terpenoids, namely neriifolins A, B, and C, from the leaves of *Euphorbia neriifolia* Linn **(26-28)**. Their cytotoxicity against breast cancer cell lines MCF-7 and MDA-MB-231 was assessed using the MTT test. Their activity against MCF-7 cells was more potent than against MDA-MB-231 cells, with IC_50_ values of 13.14 ± 1.12, 7.12 ± 0.85, and 9.50 ± 0.98 µM against MCF-7 cells, respectively. The IC_50_ values against MDA-MB-231 cells were 27.71 ± 1.44, 21.12 ± 1.33, and 26.77 ± 1.43 µM. The newly identified compounds have selective activity on MCF-7 estrogen receptor-positive cells, while not affecting MDA-MB-231 cells (Choodej and Pudhom 2020).

An extensive analysis of the ethyl acetate extract from the arial parts of *Caragana sukiensis* (a plant belonging to the Fabaceae family) was conducted to identify and characterize its chemical components. Two novel cycloartane type triterpenoids were isolated as a result of the work; 20(*R*),24(*S*)-epoxycycloartane-3*ꞵ*,6*α*,16*ꞵ*,25-tetraol-3*ꞵ*-*O*-D-(2-*O-*acetyl) xylopyranoside **(29)** and cyclosiversigenin **(30)**, as well as one new ursan type triterpene; soyasapogenol B **(31)**. The isolated compounds showed promising cytotoxic effects with IC_50_ values of 2.931, 1.778, and 2.328 µm, respectively, compared with Doxorubicin (IC_50_=1.117 µm). In addition, the flow cytometric analysis demonstrated that cyclosiversigenin induces cell apoptosis signaling via arresting cell cycle at G0/G1 phase. Moreover, the apoptotic action of cyclosiversigenin was validated using Hoechst 33258 staining, Annexin V-FITC test, and assessment of mitochondrial membrane potential (Reddy et al. 2017).

Figure 5 shows the chemical structures of triterpenoids that have been identified and could potentially be cytotoxic to breast cancer cells (**32-44**).

Rhizomes of *Alisma orientale* (Sam.) Juzep (Alismataceae) is widely used in traditional Chinese medicine and its main active constituent is a protostane triterpene named alisol A **(32)** which is investigated for its activity towards MDA-MB-231 human breast cancer cells. It effectively reduced the survival of cells in a manner that was dependent on the dose. This was achieved by triggering programmed cell death by the activation of specific proteins, including cleaved caspase 3, cleaved caspase-9, Bcl 2, and p p38. Furthermore, the administration of alisol A at a concentration of 10 µM resulted in alterations in the expression of cyclin A and cyclin D1, which served as a signal of cell cycle arrest in the G1 phase. In addition, the presence of LC3 II indicated the occurrence of autophagy through the production of reactive oxygen species (ROS) within the cells and the induction of DNA damage in MDA MB 231 cells. In addition, the quantity of cells positive for APE1 /γH2AX /LC3 II was considerably greater compared to the negative control cells. Alisol A provoked apoptosis by inhibiting the NF-κB and PI3K/AKT/mTOR signaling pathways in MDA-MB-231 cells. Additionally, treatment with 5 µM of alisol A resulted in a 62.77±12.33% decrease in cell migration rate. The Western blotting experiment revealed a considerable downregulation of MMP-2 and MMP-9 expression levels, which were found to be associated with the anti-metastatic actions of alisol A (Lou et al. 2019a, b; Shi et al. 2020).

Betulinic acid (BA) **(33)** is a naturally occurring pentacyclic triterpene found in the bark of the birch tree *Betula papyrifera* (Betulaceae). The study demonstrated that it has a suppressive effect on the growth of breast cancer cells, specifically the MCF-7 cell lines, with an IC_50_ value of 19.06 µM (G. Zhao et al. 2007), The study revealed that it has a suppressive effect on the growth of breast cancer cells (MCF-7) with an IC_50_ value of 19.06 µM. The study showed that the activity of aerobic glycolysis in the MCF-7 breast cancer cell line was inhibited by reducing lactate production, glucose uptake, and extracellular acidification rate (ECAR). Additionally, it suppressed the expression of proteins associated with aerobic glycolysis, such as c-Myc, lactate dehydrogenase A (LDH-A), and p-PDK1/PDK1 (pyruvate dehydrogenase kinase 1). (Jiao et al. 2019). Another report by Zheng et al. showed that BA targeting glucose-regulated protein 78 (GRP78) led to inhibition of aerobic glycolysis (Zheng et al. 2019).

3-O-(*E*)-*p*-coumaroylbetulinic acid (CB) **(34)** is a triterpenoid derived from betulinic acid. It is acquired from the leaves and twigs of *Strychnos vanprukii* Craib (Loganiaceae) and *Cornus florida* L. (Cornaceae) (Chien et al. 2004; Graziose et al. 2012). The MTT assay revealed a decrease in cell viability mediated by CB in breast cancer MDA-MB-231 and T-47D cells. The IC_50_ values for these cells were 5.884 µM and 2.708 µM, respectively, within the first 24 h of treatment with -O-(E)-p-coumaroylbetulinic acid. The results indicated a cell cycle arrest of 58.8% in the G0/G1 phase. This impact was correlated with a notable decrease in the mRNA levels of cyclin D1 and cyclin-dependent kinase (CDK4) in both breast cancer cell lines. In addition, the administration with CB resulted in a decrease in the production of cyclin D1 protein in MDA-MB-231 cells after 48 h. Furthermore, it also led to an increase in the levels of p21 mRNA in both MDA-MB-231 and T-47D cells. The administration of CB therapy triggers early death in breast cancer cells, as evidenced by upregulation of cleaved caspase 3, downregulation of Bcl2 and survivin, an elevation of reactive oxygen species (ROS), and disruption of mitochondrial membrane potential. The treatment with CB dramatically decreases the activity of the Notch promoter at its IC50 by reducing the expression of the Notch-targeted genes Hes1 and Hey1 in both cancer cells (Kushwaha et al. 2020).

Two triterpenoid saponins, namely Bacopasides I and II **(35 and 36)** are major members of *Bacopa monnieri* (Plantaginaceae) (Peng et al. 2010). Bacopasides II **(36)** showed a reduction in cell viability of different breast cancer cells by MTS assay with IC_50_ values of 18 µM, 19 µM and 16 µM for MDA-MB-231, MCF7, and BT-474 cells, respectively. Bacopasides I showed an IC_50_ value of more than 50 µM. The concurrent use of two compounds exhibited a synergistic inhibitory impact on the viability and proliferation of four types of breast cancer cells, namely triple negative (MDA-MB-231), estrogen receptor-positive (T-47D and MCF-7), and human epidermal growth factor. At high concentrations, they had the ability to cause G2/M cell cycle arrest and trigger apoptosis. In addition, they markedly decreased the invasion of MDA-MB-231 cells in spheroids by 97% (Palethorpe et al. 2019).

Ginsenoside Rh2 **(37)** the major active constituent in red ginseng *Panax ginseng* (Araliaceae) showed an antiproliferative effect in MCF-7 breast cancer cells, it induces hypermethylation at a CpG site and reduced expression level of C3orf67-AS1, a novel long noncoding RNA. Additionally, the siRNA alone inhibited cell growth by up to 46%, and this inhibition was further intensified to 93% when co-treated with Rh2. The siRNA boosted early apoptosis by up to 46%, and Rh2 further enhanced it by up to 117% (Jeong et al. 2019).

Cucurbitacin B (CuB) **(38)** is a lanostane skeleton triterpene with a tetracyclic structure that is commonly found in plants of the Cucurbitaceae family, such as *Momordica charantia* L. and *Trichosanthes cucumerina* (Jia et al. 2017). Liang et al. elucidated the mechanism of action of this medication as a powerful anti-breast cancer agent by both in vitro and in vivo investigations. The MTT assay was used to conduct in vitro research on MDA‐MB‐231 and SKBR‐3 breast cancer cells. The IC_50_ values of CuB were found to be 15.89 µM and 6.177 µM for MDA‐MB‐231 and SKBR‐3 cells, respectively. CuB effectively suppressed the adherence of breast cancer cells (*P* < 0.05). The micropipette aspiration (MA) technique was used to modify the mechanical characteristics of the cells, resulting in reduced deformability of breast cancer cells due to CuB. The findings from the wound-healing and transwell assays demonstrated that the application of low dosages (10, 20, and 30 nM) of CuB resulted in a decrease in the migration and invasion of breast cancer cells when compared to the control group (Liang et al. 2019).

Another report by Dittharot et al. concerning the cytotoxicity of **(38)** revealed its hypermethylation effects and reduction of oncogenic activation of breast cancer cells with IC_50_ values of 5.07, 10.91, and 50.51 µM for MDA‑MB‑231, MCF-7, and MCF-10A cells, respectively (Dittharot et al. 2019).

Saikosaponin D (SSD), is a triterpenoid saponin derived from the roots of *Bupleurum chinense* DC. (Apiaceae), was examined for its effects on MDA‑MB-231 cells using the MTT assay. Their viability was greatly reduced in a manner that depended on the dosage, with an IC_50_ of 9.9 ± 0.24 µM. Activation of the p38 mitogen-activated protein kinase (MAPK) signaling pathway in human breast cancer MDA‑MB-231 cells led to apoptosis and the phosphorylation/activation of p38 MAPK by SSD. SSD hindered the merging of autophagosomes with lysosomes, which prevented the creation of autophagosomes and halted the process of autophagic breakdown (Fu et al. 2020).

Another triterpenoid saponin extracted from roots of *B. chinense*, saikosaponin b2 (SSb2) **(40)** was examined for its cytotoxicity towards MCF-7 breast cancer cells by MTT assay. The cell growth rate was significantly reduced compared to the control group in a dose-dependent manner (0.1, 0.2, 0.5, 1, 2, 5, 10, 20, and 50 µM) over a 48-h period. Following a 48-h treatment of MCF-7 cells with SSb2 at a concentration of 5 µM, a Western blot analysis was performed. The results demonstrated a significant reduction in the expression of c-myc and cyclin D1, both of which are involved in cell proliferation within the STAT3 signaling pathway, as compared to the control group (*P* < 0.05). In addition, it decreased the levels of vasodilator-stimulated phosphoprotein (VASP), matrix metallopeptidase (MMP) 2, and MMP9. The wound-healing assay was conducted using different concentrations (0.2, 1, and 5 µM) of SSb2. It was observed that the migration rates of MCF-7 cells decreased in a manner that was dependent on the dose (Ma et al. 2019).

Triterpenoid saponin AG8 (3*β*-O-*α*-L-rhamnopyranosyl-(1→3)-[*β*-D-xylopyranosyl-(1→2)]-*β*-D-glucopyranosyl-(1→4)-[*β*-D-glucopyranosyl-(1→2)]-*α*-L-arabinopyranosyl-16*α*-hydroxy-13, 28-epoxy-oleanane) **(41)** isolated from rhizomes of *Ardisia gigantifolia* stapf. (Primulaceae) suppressed the growth and survival of various types of triple-negative breast cancer cells (TNBC), namely MDA-MB-231, BT-549, and MDA-MB-157, by inhibiting cell proliferation and inducing apoptosis. The IC_50_ values for these cell lines were 3.80, 0.73, and 1.38 µM, respectively. The primary mechanism of action of AG8 in inducing cell apoptosis in TNBC cells is by modulating the levels of GSH, SOD, and MDA, which are key components of the oxidative stress pathway (Mu et al. 2020a, b).

A new protopanaxatriol-type triterpene was isolated from the roots of *Panax notoginseng* (Burk.) Chen (Araliaceae), 3*β*, 6*α*, 12*β*, 20 (*S*)-tetrahydroxydammar-24-one-25-ene **(42)**, it possesses a distinctive *α, β*-unsaturated ketene in its side chain and exhibited substantial cytotoxic effects on MCF-7 breast cancer cells, with an IC_50_ value of 16.78 ± 0.32 µM (Shang et al. 2019).

Pristimerin **(43)**, a quinone methide triterpenoid extracted from several plants included in the Celastraceae and Hippocrateaceae families (Guo et al. 2013). Pristimerin suppressed the growth of MDA-MB-231 and MDA-MB-468 cells in a way that was dependent on the dose and time of treatment. The colony formation assay findings demonstrated that pristimerin **(43)** effectively decreased the number of colonies in both cell types. The administration of Pristimerin resulted in cellular contraction, and fragmentation of nuclei, and initiated a halt in the G1 phase of the cell cycle. Additionally, it promoted programmed cell death (apoptosis) and cellular self-degradation (autophagy), which were regulated by the signaling pathways including reactive oxygen species (ROS), apoptosis signal-regulating kinase 1 (ASK1), and c-Jun N-terminal kinase (JNK) (Q. Zhao et al. 2019). Moreover, Pristimerin **(43)** demonstrated a potent anti-growth activity on the malignant cells (MCF-7 and MDA-MB-231), and MCF-7s (cancer stem cell-enriched population) with IC_50_ values ranging between 0.38 and 1.75 µM (Cevatemre et al. 2018).

Asiatic acid (AA) **(44)** is a pentacyclic triterpenoid acid derived from the roots of *Actinidia valvata* Dunn (Actinidiaceae), a plant rich in polyphenols. The compound suppressed the function of MDA-MB-231 cells and notably elevated the expression of WAVE3 in ductal carcinoma in situ tissue. In addition, the administration of Asiatic acid at a dose of 50 *µ*M resulted in notable effects on cell apoptosis and invasion, as well as a considerable inhibition of the expression of WAVE3, P53, p-PI3K, p-AKT, and various other proteins. Furthermore, the cellular levels of WAVE3 mRNA and protein exhibited a substantial rise after the introduction of the plasmid (Gou et al. 2020).

Figure 6 shows the chemical structures of triterpenoids that have been identified and could potentially be cytotoxic to breast cancer cells (**45-61**).

*Actinidia chinensis* Planch (Actinidiaceae) is a well-known plant to treat different types of cancers in traditional Chinese medicine (Fang et al. 2016). The phytochemical investigation of 95% ethanol extract of *Actinidia chinensis* roots revealed the characterization of a new triterpenoid named 2*α*, 3*α*, 23, 24-tetrahydroxyursa-12, 20(30)-dien-28-oic acid **(45)**. It exhibited a moderate cytotoxic effect against cultured breast cancer cell line (MCF-7) with an IC_50_ value of 45.94 ± 3.62 µM, compared to cis-diamminedichloroplatinum (DDP; IC_50_=65.30 ± 2.05 µM) as positive control (Zhang et al. 2018a, b).

Two new triterpenoids; neoabieslactone L and abiesatrine Q **(46 and 47)** were isolated from leaves of ethanol extract of *Abies faxoniana* (Pinaceae). Neoabieslactone L **(46)** demonstrated the most potent cytotoxic activity against MCF-7 with IC_50_ values of 7.5 µM, while abiesatrine **(47)** exerted moderate activity (IC_50_ values= 22.20 ± 3.30 µM), compared to Doxorubicin (2.8 ± 0.3 µM) (Viet Ho et al. 2018).

A novel tetracyclic taraxerene type triterpenoid; 2, 3-*seco*-taraxer-14-en-2, 3-bioic acid **(48)** was isolated from the acetone extract of *Euphorbia alatavica* (Euphorbiaceae). This compound showed a potent cytotoxic effect against MCF-7 with an IC_50_ value of 22.3±3.1 µM, compared to the standard drug (Doxorubicin; IC_50_=15.7 ± 4.2 µM). Further, another isolated triterpenoid, named 3-hydroxy-4, 4, 8,14-tetramethyl-18-norpregnan-20-one **(49)** demonstrated the most potent cytotoxic effect (IC_50_= 8.6 ± 3.5 µM), which is better than doxorubicin (Rozimamat et al. 2017).

A new dammarane-type triterpene named vibusambucin A **(50)** was isolated from leaves of *Viburnum* sambucinum (Adoxaceae) exhibited potent inhibitory effect on MCF-7 with *an* IC_50_ value of 5.7 µM . Another two-known isolated octanor-dammarane derivatives namely, 12*β*-hydoxy-3,15-dioxo-20, 21, 22-23, 24,25, 26, 27-octanordammanran **(51)** and hupehenol A **(52)** were more cytotoxic on the same cell line with IC_50_ values of 5.30 and 5.10 µM, respectively (Nguyen et al. 2017).

Katononic acid **(53)**, an oleanene-type triterpenoid, was isolated from the *n*-hexane fraction of *Nuxia oppositifolia* (Stilbaceae) aerial parts. It showed a potent cytotoxic effect on the MDA-MB-231 breast cancer cell line with an IC_50_ value of 18.25 µM, compared to Doxorubicin (IC_50_= 15.41 µM) (Al-Massarani et al. 2017).

Phytochemical characterization of sweet chestnut heartwood *Castanea sativa* (Fagaceae) revealed the presence of new ursane-type triterpenoid saponins. It was found that chestnoside B **(54)** was the most potent cytotoxic drug against MCF-7 cells with an IC_50_ value of 12.3 µM, compared with Oxaliplatin (12.8 ± 2.8 µM) and Cisplatin (16.7 ± 3.5 µM) (Pérez et al. 2017).

Soulieoside O **(55)**, a new cyclolanostane triterpenoid glycoside, was isolated from the 95% ethanol extract of *Souliea vaginata* rhizomes (Ranunculaceae). The new compound displayed a potent inhibitory activity against MDA-MB231 breast cancer cell line with an IC_50_ value of 9.3±2.10 µM. In addition, 5-flurouracil was used as the positive control (IC_50_ = 87.6 ± 4.9 µM) (Wu et al. 2017).

Five new oleanane-type saponins, hirsutosides A-E, were characterized as the major compounds of *Glochidion hirsutum* (Phyllanthaceae) leaves and screened for cytotoxic effect using the SRB assay. Among these compounds, hirsutosid B **(56)** was identified as the most promising cytotoxic (IC_50_*=* 4.7 ± 0.6 mM) against MCF-7, compared with Ellipticine (2.0 ± 0.3 mM) (Thang et al. 2017).

Tingenin b (57), a quinone-methide triterpenoid, was isolated from the root bark of *Mytenus sp.* (Celastraceae) and tested against a group of breast stem cells enriched from the MCF-7 cell line. According to reports, tingenin b exhibited cytotoxic effects on MCF-7 cells, with an IC_50_ value of 2.38 µM. From a mechanistic standpoint, it was clear based on the positive staining of Annexin V, the decrease in mitochondrial membrane potential, and the dephosphorylation of Bcl-2, that there was a simultaneous rise in the expression of Bax protein. Furthermore, it was discovered that tingenin b-induced cell death is associated with endoplasmic reticulum stress (Cevatemre et al. 2016).

Schisanlactone B **(58)** a triterpenoid isolated from the stems of *Kadsura coccinea* (Schisandraceae), it showed potent cytotoxicity against MDAMB-231 cancer cell lines with IC_50_ values of 2.38 ± 0.02 µM as compared to Adriamycin used as a positive control with IC_50_ values of 0.08 ± 0.01 µM (Tram et al. 2021).

New cytotoxic tetracyclic triterpenoids were isolated from roots and aerial parts of *Monadenium lugardae* (Euphorbiaceaewas), namely lugardstatin 1 **(59)** and lugardstatin 2 **(60)**. They showed potent cytotoxicity against MCF-7 with IC_50_ values of 0.79 and 0.68 µM, respectively (Pettit et al. 2016).

From the air-dried roots of *Leplaea mayombensis* (Meliaceae) leplaeric acid B (3-methyl ester of leplaeric acid A) **(61)** was isolated and identified as a seco-lanostane-type triterpenoid. It showed cytotoxic activity against the MDA MB231 cell line with an IC_50_ value of 55.0 ± 7µM (Sidjui et al. 2017).

Figure 7 shows the chemical structures of triterpenoids that have been identified and could potentially be cytotoxic to breast cancer cells (**62-71**). Secofriedelanophyllemblicine **(62)** was isolated from the root of *Phyllanthus emblica* L. (Phyllanthaceae) and studied for its antiproliferative properties. It demonstrated a significant decrease in cell viability of MCF-7 breast cancer cells by 70.65% when treated with 2 µM. In addition, using flow cytometric examination, it was observed that the compound suppressed cell growth and caused a halt in the G2 phase of the cell cycle in MCF-7 cells (Wang et al. 2022a, b).

Endertiin A **(63)**, a lanostane triterpenoid, was derived from the fruit bodies of the mushroom *Humphreya endertii*. Its cytotoxic effects on the MCF-7 cell line were assessed. Endertiin A **(63)** demonstrated significant growth suppression of MCF-7 cells, with an IC_50_ value of 71.16 ± 6.25 µg/mL (Quang et al. 2022).

Corosolic acid **(64)**, chemically referred to as 2*α*-hydroxyursolic acid, is a type of pentacyclic triterpenoid that is found in *Lagerstroemia speciosa*, which belongs to the Lythraceae family. Exposing the MADA-MB-231 and MCF7 cell lines to corosolic acid resulted in the stimulation of apoptosis-related caspases, specifically Caspase-8, 9, and -3, in MADA-MB-231 cells. However, there was no impact on apoptotic markers in MCF7 cells. The apoptosis of MADA-MB-231 cells was triggered by corosolic acid through the reduction of phosphorylated JAK2 and STAT3 protein levels. The viability of the MDA-MB-231 cell line was considerably decreased by corosolic acid at a dose of 15 µM and higher, with an IC_50_ value of 20.12 µM. The IC_50_ value for the antiproliferative impact of corosolic acid on the MCF7 cell line after 48 hours was determined to be 28.50 µM (Jasim et al. 2023).

Lasiocarpine A **(65)** was isolated from the fruits of *Chisocheton lasiocarpus* (Meliaceae). Its cytotoxic activity against the breast cancer cell line MCF-7 was assessed using the PrestoBlue reagent. The compound had the highest level of activity, as shown by an IC_50_ value of 43.38 µM (Katja et al. 2023).

## In vivo studies of plant triterpenoids on the management of breast cancer

**Fig. 8:**
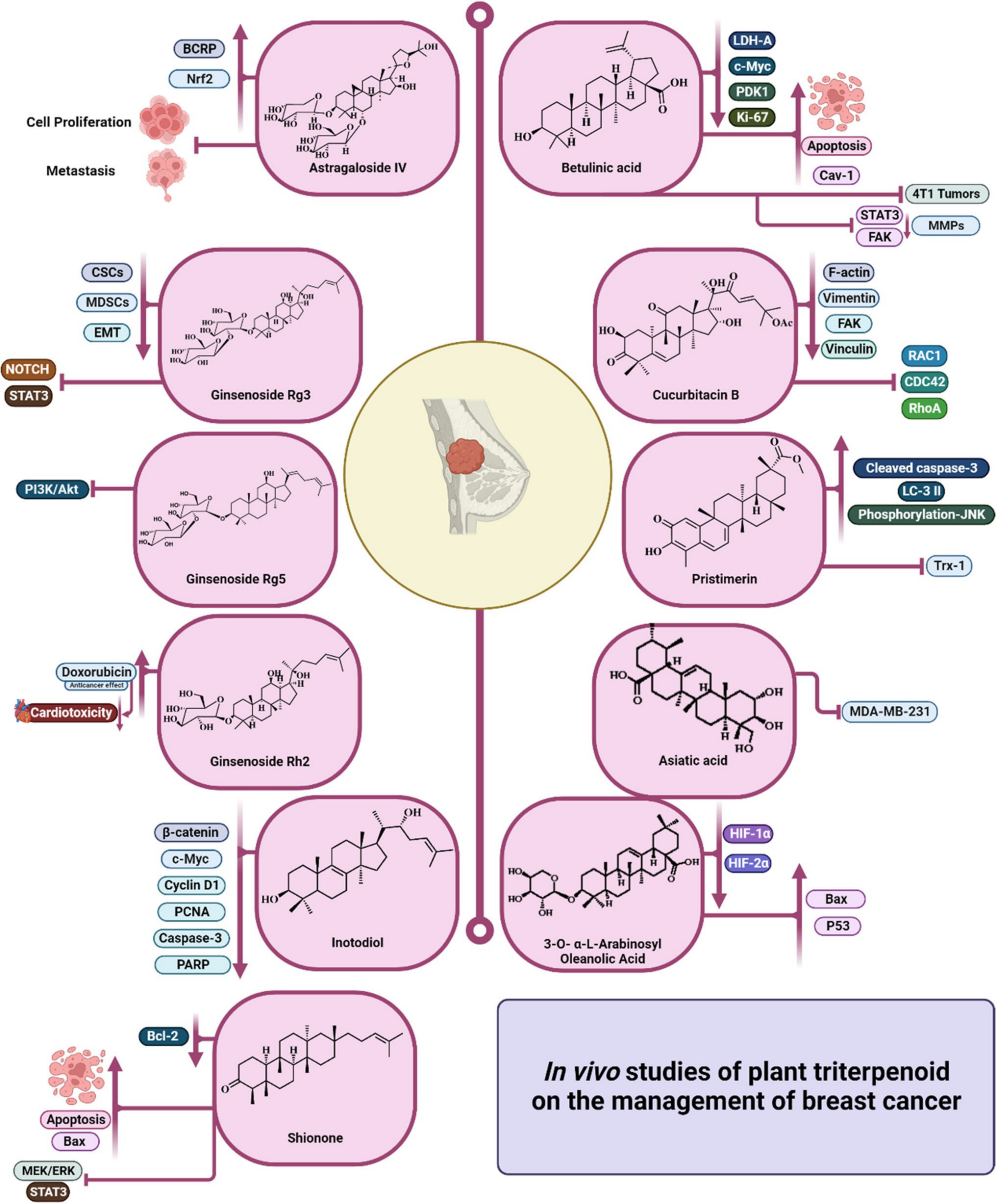
The triterpenoids with potential *in vivo* cytotoxicity towards breast cancer cell lines

The triterpenoids that have been shown to have potential *in-vivo* cytotoxicity towards breast cancer cell lines are illustrated in **(**Fig. 8**)**.

Betulinic acid **(33)**, a pentacyclic triterpenoid compound, is characterized by different *Betula* species (Liu et al. 2016). The anti-cancer effects of BA were confirmed through *in-vivo* investigations conducted on transgenic MMTV-PyVT+/- female mice, which are prone to developing mammary tumors, as well as on a zebrafish model of breast cancer xenotransplantation. Administration of BA through intraperitoneal injection at a dosage of 250 mg/kg resulted in notable suppression of breast cancer growth and glycolytic activity in the transgenic MMTV-PyVT+/- spontaneous breast cancer model. This was accompanied by a significant decrease in the expression of glycolysis-related proteins such as LDH-A, c-Myc, and PDK1. Additionally, the proportion of cancer cells undergoing apoptosis was significantly higher compared to the control group. Furthermore, there was a decrease in the expression of the essential cancer proliferation marker Ki67. Furthermore, tumor samples treated with BA exhibited an increase in Cav-1 expression and a notable decrease in c-Myc and PDK1 levels in tumor tissues, providing evidence that a dosage of 250 mg/kg BA can effectively suppress glycolytic activity in vivo. When BA was given at concentrations higher than 20 µM in the zebrafish breast cancer xenotransplantation model, it effectively suppressed the growth of the transplanted MCF-7 cells in zebrafish. Furthermore, the immunohistochemical experiment demonstrated that BA not only increased the expression of Cav-1 but also reduced the expression of c-Myc in the transplanted MCF-7 cells in zebrafish (Jiao et al. 2019).

Furthermore, the anti-metastatic efficacy of betulinic acid **(33)** was assessed in both the 4T1 tumor model and the 4T1 caudal vein model in BALB/c mice. It was discovered that betulinic acid reduced the capacity of three types of breast cancer cells (MCF-7, 4T1, and MDA-MB-231) to survive, and this effect was dependent on the concentration of the acid. Moreover, it can impede the activation of stat3 and FAK, resulting in a reduction in matrix metalloproteinases (MMPs) and an augmentation in the synthesis of MMPs inhibitor (TIMP-2).

Additionally, the administration of betulinic acid at a dosage of (10 mg/kg/day, i.p.) effectively inhibited the growth of 4T1 tumors and prevented the development of lung metastases, all while exhibiting no adverse effects. The histological and immunohistochemical analyses revealed the presence of activated cleaved caspase-3 positive cells and a limited population of myeloid-derived suppressor cells (MDSCs) in both the lungs and malignancies. Jiao and his team published a study that found botulin can restrict aerobic glycolysis in MCF-7 and MDA-MB-231 cells. This is achieved by reducing lactate production, glucose uptake, extracellular acidification rate (ECAR), and inhibiting proteins related to aerobic glycolysis, such as c-Myc, lactate dehydrogenase A (LDH-A), and p-PDK1/PDK1 (pyruvate dehydrogenase kinase 1) (Jiao et al. 2019). Furthermore, it enhances the activity of the Cav-1/NF-κB/c-Myc pathway in both the transgenic MMTV-PyVT breast cancer spontaneous model and the zebrafish breast cancer xenotransplantation model, without any reported adverse effects (Zeng et al. 2018).

Liang et al. conducted an additional investigation to examine the impact of Cucurbitacin B (CuB) **(38)** on the migration of breast cancer cells *in vivo* using a mouse lung metastasis model. The findings demonstrated that CuB efficiently inhibited cell adhesion and deformability, while also modifying the viscoelastic properties of breast cancer cells. It had the capacity to hinder the movement and infiltration of breast cancer cells. Regarding the underlying mechanisms, it caused a decrease in the expression of F-actin, vimentin, FAK, and vinculin, leading to changes in the distribution and rearrangement of cytoskeletal proteins in breast cancer cells. Additionally, it suppressed the activity of GTPases RAC1, CDC42, and RhoA which are involved in integrin signaling pathways. In addition, CuB caused less inflammation and fewer negative effects compared to vincristine. Through the use of an immunofluorescence test, CuB was found to reduce the intensity of F-actin/vimentin/FAK/vinculin in breast cancer cells. This resulted in changes to the distribution and arrangement of cytoskeletal proteins within the cells, ultimately suppressing their ability to adhere, migrate, and invade (Liang et al. 2019).

Further investigation on the quinine methide triterpenoid pristimerin **(43)** activity *in vivo* was evaluated based on its ability to suppress the proliferation of MDA-MB-231 tumor xenografts in nude mice. The western blot analysis of the tumor tissues revealed that pristimerin increased the levels of cleaved caspase-3, LC-3 II, and phosphorylation-JNK. Additionally, it suppressed the activity of Trx-1 in the tumors. These findings align with the results obtained from *in-vitro* investigation (Zhao et al. 2019). Also, at lower levels (<1.56 µM), it hindered the formation of spheres in NOD by triggering apoptosis and autophagy. CB17-Prkdcscid/J mice (Cevatemre et al. 2018).

Various doses of asiatic acid **(44)** were administered orally on 14 consecutive days. A xenograft tumor model was established in immunodeficient mice by inoculating them with the human breast cancer cell line MDA-MB-231. The findings demonstrated that the administration of asiatic acid at a dosage of 50 mg/kg effectively suppressed the growth of MDA-MB-231 xenografted tumors in nude mice, resulting in a tumor inhibitory rate of 59.55% (Gou et al. 2020).

Astragaloside IV (AS-IV) **(66)** is a compound that has been isolated from the dried roots of *Astragalus membranaceus* Fisch. (Fabaceae). An investigation was conducted to assess its *in vivo* effect on breast cancer resistance protein (BCRP). The expression of BCRP was upregulated and the expression of Nrf2 was considerably increased in the liver of wild-type mice. In addition, AS-IV significantly raised the activity of ARE-luciferin and the translocation of Nrf2 to the nucleus in cells. It also increased the efflux activity of P-gp and BCRP, and elevated intracellular ATP levels (Lou et al. 2019a, b). Another report by Hu et al. revealed that AS-IV inhibits cell proliferation and metastasis of breast cancer through the promotion of the long noncoding RNA TRHDE‑AS1 where the Low expression of TRHDE-AS1 is linked to poor outcomes in breast cancer patients and plays a role in the aggressive tumor biology of breast cancer (Hu et al. 2021).

A study was conducted to investigate the anti-cancer properties of ginsenoside Rg3 **(67)**, a natural panax triterpenoid saponin, in vivo using a mouse tumor model with FM3A breast carcinoma cell-derived tumors. Administration of Rg3 resulted in a substantial decrease in tumor growth in comparison to the control group. Additionally, it has the ability to efficiently suppress the growth of breast tumors through various mechanisms, such as reducing the activity of cancer stem-like cells (CSCs) and myeloid-derived suppressor cells (MDSCs) that promote cancer stemness, inhibiting epithelial-mesenchymal transition (EMT), blocking the STAT3-dependent pathway, suppressing tumor-derived cytokines, and interfering with the NOTCH signaling pathway (Song et al. 2020).

Ginsenoside Rg5 **(68)**, a naturally occurring compound found in black ginseng (Araliaceae), was examined for its potential to inhibit breast cancer in a mouse model of human breast cancer using BALB/c nude mice. The tumor growth rate was reduced by 71.40% at a dosage of 20 mg/kg, with no noticeable adverse effects. This reduction was compared to the inhibition rate of 72.0% observed with docetaxel. The compound triggered programmed cell death and self-degradation processes in breast cancer tissues. This was achieved by activating specific pathways involved in cell death and cellular waste removal. The compound stimulated the death receptor pathway and the mitochondrial signaling pathway within the cells. Additionally, it promoted the formation of autophagosomes and the accumulation of specific proteins involved in autophagy. Furthermore, it inhibited the PI3K/Akt signaling pathway (Liu and Fan 2018).

In a further study performed by Hou et al., it was observed that ginsenoside Rh2 **(37)** demonstrated strong effectiveness in treating breast cancer in mice that were also receiving doxorubicin. The study found that doses of 20 and 30 mg/kg of ginsenoside Rh2 considerably improved the anticancer effects of doxorubicin while simultaneously reducing cardiotoxicity during the treatment period (Hou et al. 2022)

An edible medicinal mushroom (*Inonotus obliquus*) has been utilized in traditional medicine to treat different human diseases (Shashkina et al. 2006). A lanostane triterpenoid namely, inotodiol **(69)** was obtained as the major compound from *Inonotus obliquus*. An *in vivo* study was conducted in a female Sprague-Dawley rat model of diabetic breast cancer. The study found that inotodiol **(69)** effectively inhibited the growth of breast cancer cells by triggering programmed cell death, known as apoptosis. The study found that it had the potential to decrease the expression of β-catenin and its downstream targets (c-Myc and Cyclin D1) in rat mammary tissues induced by STZ-DMBA. In addition, immunohistochemistry staining for PCNA verified that the drug decreased the expression of the tumor proliferation marker PCNA. Furthermore, it decreased the levels of caspase-3 and poly (ADP-ribose) polymerase (PARP) gene expressions (Zhang et al. 2018a, b).

Shionone **(70)** a natural triterpenoid isolated from roots of *Aster tataricus* Fam. Asteraceae (Wang et al. 2012). The transwell tests and western blot analysis demonstrated that the treatment resulted in the inhibition of cell proliferation, migration, and invasion in both SK-BR-3 and MB-157 breast cells. This was achieved through the activation of apoptosis and the suppression of the MEK/ERK and STAT3 signaling pathways. Shionone induced the splitting of caspase-3 and 9. Shionone resulted in an upregulation of Bax expression and a downregulation of Bcl-2 expression (Xu et al. 2020).

From the leaves of *Schumacheria castaneifolia* Vahl. (Dilleniaceae) a triterpenoid saponin 3-O-*α*-L-arabinosyl oleanolic acid (3-O-L-AO) **(71)** isolated and showed antiproliferative effects in breast cancer stem cells (bCSCs) maintained in low oxygen conditions, as determined by the WST-1 assay. The results demonstrated a notable increase in the expression of Bax and p53, and a notable decrease in the expression of survivin, HIF-1α, and HIF-2α (Muthoni et al. 2021).

The triterpenoids that have been shown to have potential *in vivo* cytotoxicity towards breast cancer are outlined in (Table 2).

**Table 2:** Plant triterpenoids with potential in vivo inhibitory activity against to breast cancer

Compound name	Dosage	Animal model	Findings	Ref.
Betulinic acid (33)	10 mg/kg/day, *i.p.*	Transgenic MMTV-PyVT+/- female mice, which are prone to developing mammary tumors	-Significant inhibition of cancer tumor -Significant decrease in the expression of glycolysis-related proteins such as LDH-A, c-Myc, and PDK1 -Decrease in the expression of the essential cancer proliferation marker Ki67.	Liu et al. (2016).
	10 mg/kg	4T1 Caudal vein model in BALB/c mice	-Reduction the capacity of MCF-7, 4T1, and MDA-MB-231) in a concentration- dependent manner. -impede the activation of stat3 and FAK. -A reduction in MMPs -Augmentation of TIMP-2 synthesis. -Anti-metastatic effect	Jiao et al. (2019)
	250 mg/kg	A zebrafish model of breast cancer xenotransplantation.	-A notable suppression of breast cancer growth and glycolytic activity -Induction of apoptosis -Increase in Cav-1 expression and a notable decrease in c-Myc and PDK1 levels in tumor tissues -Suppression the growth of the transplanted MCF-7 cells in zebrafish.	Jiao et al., (2019). Zeng et al. (2018)
Ginsenoside Rh2 (37)	20 and 30 mg/kg	Mice receiving doxorubicin	- Improvement of the anticancer effects of doxorubicin -Reduction of cardiotoxicity	Hou et al. (2022)
Cucurbitacin B (38)	100nM	A mouse lung metastasis model	-Inhibition of cell adhesion and deformability -Modification of the viscoelastic properties of breast cancer cells -Hinder the movement and infiltration of breast cancer cells -Down-expression of F-actin, vimentin, FAK, and vinculin -Changes in the distribution and rearrangement of cytoskeletal proteins in breast cancer cells. -Suppression in the activity of GTPases RAC1, CDC42, and RhoA.	Liang et al. (2019)
Pristimerin (43)	100 µL	Tumor xenografts in nude mice.	-Suppression of the proliferation of MDA-MB-231. -Increase in the levels of cleaved caspase-3, LC-3 II, and phosphorylation-JNK -Suppression of Trx-1 activity in the tumors	Zhao et al. (2019)
	1.56 µM	CB17-Prkdcscid/J mice	-Hinder the formation of spheres in NOD -Triggering apoptosis and autophagy.	Cevatemre et al. (2018)
Asiatic acid (44)	50 mg/kg	Xenograft tumor model in nude mice	-Suppression of MDA-MB-231 xenografted tumor growth -A tumor inhibitory rate=59.55%	Gou et al. (2020)
Astragaloside IV (66)	20 mg/kg	Wild-type mice bearing breast cancer resistance protein (BCRP).	-Upregulation of BCRP expression. -Over-expression of Nrf2 -Increase of ARE-luciferin activity -Translocation of Nrf2 to the nucleus in cells -Increase of the efflux activity of P-gp and BCRP, and elevated intracellular ATP levels	Lou et al. (2019a, b)
	20 mg/kg	BALB/c nude mice bearing TRHDE-AS1-silenced MDA-MB-231cells	-Inhibition of breast cancer cell proliferation and metastasis -Promotion of the long noncoding RNA TRHDE‑AS1 where the Low -Expression of TRHDE-AS1 was linked to poor outcomes in breast cancer patients	Hu et al. (2021).
Ginsenoside Rg3 (67)	3, 6, 12.5, 25 µg/mL	A mouse tumor model with FM3A breast carcinoma cell-derived tumors.	-Substantial decrease in breast tumor growth -Reduction of the activity of CSCs and MDSCs -Inhibition of EMT, blocking the STAT3-dependent pathway. -Suppressing tumor-derived cytokines, and interfering with the NOTCH signaling pathway	Song et al. (2020)
Ginsenoside Rg5 (68)	20 mg/kg	A mouse model of human breast cancer using BALB/c nude mice	-The tumor growth rate was reduced by 71.40% -The inhibition rate = 72.0% - Triggering programmed cell death and self-degradation processes in breast cancer tissues. -Stimulation of the death receptor and the mitochondrial signaling pathways. -Promotion of the formation of autophagosomes and the accumulation of specific proteins involved in autophagy. -Inhibition of the PI3K/Akt signaling pathway	Liu and Fan (2018)
Inotodiol (69)	10 mg/kg	A female Sprague-Dawley rat model of diabetic breast cancer (STZ-DMBA).	-Inhibition of the growth of breast cancer cells --Induction of apoptosis. -Decrease of the expression of β-catenin and its downstream targets (c-Myc and Cyclin D1) in rat mammary tissues. -Decrease of tumor proliferation marker PCNA expression. Down-expression of caspase-3 and poly (ADP-ribose) polymerase (PARP) gene.	Shashkina et al. (2006), X. Zhang et al. (2018a, b)

## Future perspectives

The plant’s triterpenoids have shown significant *in vitro* and *in vivo* biological properties regarding breast cancer. This research should be expanded to determine the potential for additional application in the industry. Future research should conduct further investigations to elucidate the pharmacodynamic and pharmacokinetic aspects associated with the identified biological effects. Additionally, it is necessary to conduct molecular docking of the triterpenoids found in plants to ascertain the specific target proteins associated with their biological capabilities. The utilization of plant triterpenoids in manufacturing processes has prompted interest in patented products in order to encourage their integration and commercialization in the pharmaceutical industry. Furthermore, a thorough examination of biologic studies implementing mechanistic techniques, as well as pre-clinical and toxicological investigations, is necessary. Another suggested approach involves employing various techniques to enhance the production of terpenoids in the plant. Future studies should prioritize the evaluation of stability and maintenance of triterpenoids’ biological activity, particularly over extended storage periods. This is crucial to advance towards bigger production scales and the development of industrial products.

## Conclusions

The current review successfully confirmed the positive impact of triterpenoids on breast cancer and provides their mechanism of action on different breast cancer cells in both assays *in vitro* and *in vivo* rendering them an attractive candidate for the production of functional drugs in the pharmaceutical industry. Nevertheless, the review provides a foundation for further investigation of triterpenoids in randomized, placebo-controlled clinical trials to demonstrate their therapeutic efficacy in the clinical setting. Finally, standardization of the drug is a demand to be accepted in conventional medicine. Further investigations are necessary to determine the advisability of consuming triterpenoids as a component of breast cancer treatment and control. Furthermore, it is necessary to develop more breast cancer cell/tumor models for both laboratory and animal testing, as well as conduct additional preclinical studies, to establish the most effective dosage for therapeutic purposes.

## Data Availability

Not applicable.
